# Stepwise observation and quantification and mixed matrix membrane separation of CO_2_ within a hydroxy-decorated porous host†
†Electronic supplementary information (ESI) available. CCDC 1504685–1504693. See DOI: 10.1039/c6sc04343g


**DOI:** 10.1039/c6sc04343g

**Published:** 2017-02-27

**Authors:** Christopher G. Morris, Nicholas M. Jacques, Harry G. W. Godfrey, Tamoghna Mitra, Detlev Fritsch, Zhenzhong Lu, Claire A. Murray, Jonathan Potter, Tom M. Cobb, Fajin Yuan, Chiu C. Tang, Sihai Yang, Martin Schröder

**Affiliations:** a School of Chemistry , University of Manchester , Oxford Road , Manchester , M13 9PL , UK . Email: Sihai.Yang@manchester.ac.uk ; Email: M.Schroder@manchester.ac.uk; b Diamond Light Source , Harwell Science and Innovation Campus , Didcot , Oxfordshire , OX11 0DE , UK . Email: chiu.tang@diamond.ac.uk; c Department of Chemistry , University of Liverpool , Liverpool , L69 7ZD , UK; d Fraunhofer IAP , FB3, Geiselbergstrasse 69 , Potsdam-Golm , 14476 , Germany

## Abstract

The identification of preferred binding domains within a host structure provides important insights into the function of materials. State-of-the-art reports mostly focus on crystallographic studies of empty and single component guest-loaded host structures to determine the location of guests. However, measurements of material properties (*e.g.*, adsorption and breakthrough of substrates) are usually performed for a wide range of pressure (guest coverage) and/or using multi-component gas mixtures. Here we report the development of a multifunctional gas dosing system for use in X-ray powder diffraction studies on Beamline I11 at Diamond Light Source. This facility is fully automated and enables *in situ* crystallographic studies of host structures under (i) unlimited target gas loadings and (ii) loading of multi-component gas mixtures. A proof-of-concept study was conducted on a hydroxyl-decorated porous material MFM-300(V^III^) under (i) five different CO_2_ pressures covering the isotherm range and (ii) the loading of equimolar mixtures of CO_2_/N_2_. The study has successfully captured the structural dynamics underpinning CO_2_ uptake as a function of surface coverage. Moreover, MFM-300(V^III^) was incorporated in a mixed matrix membrane (MMM) with PIM-1 in order to evaluate the CO_2_/N_2_ separation potential of this material. Gas permeation measurements on the MMM show a great improvement over the bare PIM-1 polymer for CO_2_/N_2_ separation based on the ideal selectivity.

## Abstract 2

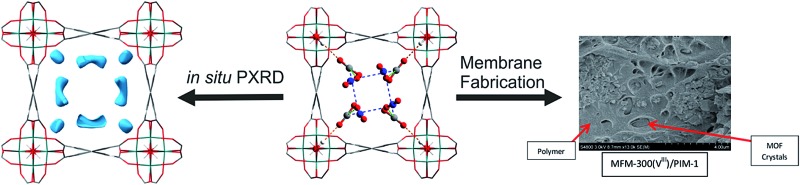
CO_2_ binding and separation using porous MFM-300(V^III^) has been fully studied.

## 1. Introduction

With increasing levels of anthropogenic CO_2_ emissions contributing to climate change, there is growing research interest in the field of carbon capture and sequestration (CCS).^1^ Capture of CO_2_ from flue gas *via* selective adsorption by porous adsorbents is particularly promising because of a number of advantages over the state-of-the-art carbon capture system based upon corrosive amine scrubbers.^2^ For example, most solid adsorbents have reduced toxicity in comparison to amines, and more importantly, their operation involves a much lower energy input for regeneration due to the comparatively weaker binding interactions between the adsorbents and CO_2_.^3^ Metal–organic frameworks (MOFs) are an emerging class of porous solids showing potential in a wide range of applications such as gas adsorption and storage,^4–7^ gas separation,^8,9^ catalysis,^10,11^ drug delivery,^12^ and sensing.^13^ MOFs, constructed by metal ions/clusters bridged by organic linkers, usually show crystalline extended structures with high internal surface areas and pore volumes. Within the field of gas adsorption, the identification of preferred binding sites at a molecular level within the MOF structure represents an important methodology for understanding the host–guest (MOF–gas) binding interactions and any observed selectivity. However, such studies are often very challenging because most porous adsorbents (*e.g.*, activated carbons, porous silica, and large pore zeolites) lack structural order. In contrast, the highly crystalline nature of MOFs makes them particularly advantageous in this perspective since it enables the interrogation of preferred binding domains *via* advanced crystallographic investigations.^3,8,9,14–22^

Although single crystal diffraction is the most powerful crystallographic technique to study the location of guests, full retention of single crystalline MOF samples upon desolvation at elevated temperatures, and with sequential guest loading under pressure is only achieved in exceptional cases.^14–19^ On the other hand, techniques such as powder X-ray diffraction (suitable for CO_2_, SO_2_) and neutron powder diffraction (suitable for H_2_ and hydrocarbons) are also being employed to probe *in situ* host–guest structures.^3,8,20–25^ However, most of the current experimental systems suffer from two major limitations. Firstly, precise control of gas loading can be highly problematic in manual operation, not least because it is time-consuming, and as a result, most of the studies rely on the characterisation of the bare and gas-loaded structures under one or two pressures (typically atmospheric pressure). Information on the gas uptake process over a wide pressure range is therefore lost. Secondly, precise dosing with mixtures of gases at desirable and variable ratios that reflect more closely real world conditions is difficult to achieve and thus molecular details on the competitive binding between two different gas species in the same pore environment are unclear. Previously, a manually operated system was used to perform such studies on the high resolution powder diffraction beamline I11 at Diamond Light Source.^26^ This system, however, suffers from the aforementioned issues regarding precise control, and is also time-consuming to operate because of the stringent “search activity” at synchrotron beamlines each time the hutch is opened for researcher access. Therefore, the development of programmable equipment to facilitate these studies with greater precision is essential. Automation of operations will afford more efficient use of beam time at such facilities by increasing sample capacity and turnover at gas different pressures. Herein we present the design and construction of a new remote control gas dosing system. When coupled with the diffraction beamline, it enables advanced crystallographic studies on the host materials with (i) unlimited single-component gas loadings with precise control and (ii) loadings of multicomponent gas mixtures at desirable ratios and pressures. More significantly, all of these operations and data collections can be carried out by automated computer programme without the need for researcher access to the beamline (except from setting up the sample at the beginning). A proof-of-concept study was conducted on a hydroxyl-decorated porous material MFM-300(V^III^) (i) under five different CO_2_ pressures covering fully the isotherm range and (ii) on loading of an equimolar mixture of CO_2_ and N_2_ at different pressures. This study has captured successfully the details of the structural dynamics underpinning CO_2_ uptake over the entire surface coverage, and identified CO_2_ binding at crystallographic resolution. The results provide direct structural evidence for the observed high CO_2_/N_2_ adsorption selectivity for MFM-300(V^III^).

Investigation of the separation selectivity of a given material under dynamic flow conditions can afford critical insights beyond the static isotherm selectivity. A potential method for utilising MOFs in CCS, whilst simultaneously reducing the financial impact of the current high cost of MOF production, is incorporating them in a mixed matrix membrane (MMM).^27,28^ MMMs are composite materials combining a polymeric matrix with an inorganic filler and has attracted significant attention in recent years although the concept has been known since the late 1980s, where components such as aluminosilicates and CaY zeolites were added to organic polymers to form films for separation of gases^29^ and saccharides.^30^

A membrane which exhibits the ideal properties for gas separation will have high adsorption selectivity but simultaneously having ultrahigh permeability to assist the gas diffusion. However, there is an inherent trade-off between selectivity and permeability as represented by the Robeson upper bound.^31,32^ Initially set in 1991, this upper bound demonstrated the trade-off between the two factors affecting the efficacy of these membranes.^31^ As membrane technology has evolved, this upper bound was revised in 2008.^32^ The majority of pure polymeric membranes are unable to exceed the upper bound limit.^32^ Inorganic membranes have been shown to have very good selectivity and permeability and therefore exceed the upper bound. However, they have been shown to be poorly reproducible, expensive and mechanically unstable.^33^ In contrast, MMMs show a good compromise between pure organic and inorganic polymer membranes and are considered to be promising candidates to fall above the Robeson upper bound whilst being more reproducible and mechanically stable. Although initial studies on MMMs focussed on zeolites,^34^ recent attention has turned to MOFs due to the increased affinity shown between the MOF particles and the polymer matrix.^35^ This increased affinity is due to the hybrid inorganic–organic nature of MOFs and has been shown to lead to fewer non-selective macro-voids at the interface between the MOF particles and polymer matrix, as commonly seen in zeolite-based MMMs.^34^ Such voids allow unrestricted gas permeation and dramatically decrease the separation capability of the MMM. Herein, we show that MFM-300(V^III^) can be incorporated in a MMM with PIM-1 without generating macro-voids. Dynamic gas permeation measurements on MFM-300(V^III^)/PIM-1 have shown great improvement over the bare PIM-1 polymer for CO_2_ separation based on the ideal selectivity.

## 2. Experimental

### 2.1. Design and construction of the automated gas dosing system at Diamond powder diffraction Beamline I11

**Fig. 1:**
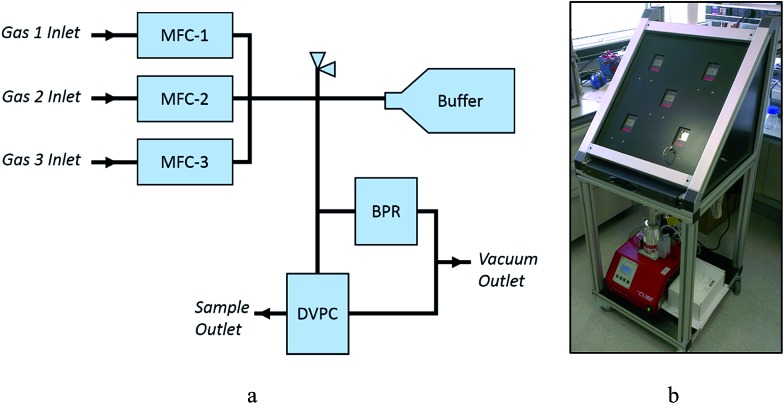
(a) Schematic and (b) photographic views of the remote controlled gas dosing system at Diamond powder diffraction Beamline I11.

A schematic view of the automated gas dosing system is shown in Fig. 1. The system consists of three Alicat MCS-1SLPM-D mass flow controllers (MFCs), an Alicat PCS-100PSIA-D pressure controller functioning as a programmable back-pressure regulator (BPR), and an Alicat PCDS-100PSIA-D dual valve pressure controller (DVPC) connected with 6 mm stainless steel Swagelok tubing and connectors. A pressure relief valve (5.5 bar absolute) protects the system components from over-pressurisation. Two Swagelok pneumatically-activated bellows-sealed valves are used to control the venting of the system to a COSHH level to a vacuum pump. The system is compatible with a wide range of gases, including CO_2_, N_2_, air, Ar, CH_4_, CO, C_2_H_6_, H_2_, He, N_2_O, Ne, O_2_, C_3_H_8_, *n*-C_4_H_10_, C_2_H_2_, C_2_H_4_, i-C_4_H_10_, Kr, Xe, SF_6_, NO, NF_3_, NH_3_, SO_2_ and H_2_S.

To dose a sample, the target gas is first flowed through the MFCs into the main body and buffer cylinder to a pressure slightly higher than the desired final sample pressure. The sample cell is then dosed from this reservoir of gas at a programmable flow rate, with the system isolated from the gas cylinders by the MFCs. The gas dosing system has been fully integrated into the Generic Data Acquisition (GDA) software and the Experimental Physics and Industrial Control System (EPICS) at Diamond Light Source, and can be controlled either *via* EPICS GUI or GDA command line. Commands have been written for filling the system, flushing the system, and setting the sample pressure. It is important that any pressure changes that the sample is subjected to occur gradually to minimise sample movement inside the capillary. An example of the logic used in the GDA commands is shown in Fig. S1.†

### 2.2. Collection of *in situ* PXRD data for MFM-300(V^III^) upon loading of CO_2_ and equimolar mixture of CO_2_/N_2_ at different pressures

A powder crystalline sample (particle size < 0.2 μm) of MFM-300(V^III^) was loaded into a 0.7 mm borosilicate capillary which was secured into a static sample cell.^26^ The sample was activated at 393 K under dynamic vacuum (1 × 10^–8^ bar) for 2 h to remove the free solvents from the pore. The activated sample was then cooled to 273 K and PXRD data collected for 1 h with the high resolution MAC detector at Beamline I11.^36^ The bare sample was then dosed with 100, 300, 500, 750, and 1000 mbar of CO_2_ at 273 K, incrementally in 10 mbar steps to minimise the movement of the powder sample within the capillary. The gas-loaded sample was left for 30 min to achieve equilibration of the host–guest structure before PXRD data collection for 1 h at 273 K. After final data collection, the sample was re-activated at 393 K under dynamic vacuum (1 × 10^–8^ bar) for 30 min to fully remove adsorbed CO_2_ molecules. The re-activated sample was then dosed with N_2_ to 300, 500 and 1000 mbar with 15 minute data collections at 273 K with 30 min to allow equilibration for each dosing. Finally, the sample was re-activated and dosed with an equimolar mixture of CO_2_ and N_2_ to 600, 1000 and 2000 mbar with 1 h data collection at 273 K and 30 min to allow equilibration for each dosing. It is important to emphasise that all above operations and data collections (*ca.* 20 hours in total) were carried out by the programmable gas dosing and integrated data collection system without human interruption during the entire experiment.

### 2.3. Details of the structural refinement of PXRD data

Structures and unit cells of MFM-300(V^III^) over the range of loadings were refined using the Rietveld and Pawley methods, respectively, using TOPAS Academic 5. The backgrounds of the profiles were fitted using a Chebyshev polynomial with 13 coefficients, and the Stephens anisotropic peak function^37^ was used to account for the slight anisotropic line broadening. Two well-defined regions of electron density in the pore were revealed by difference Fourier analysis. In the pure CO_2_ experiment, these regions were assigned as CO_2_, and in the experiments using an equimolar CO_2_/N_2_ mixture, these regions were also assigned as CO_2_ rather than N_2_, due to both the similarity in position to the pure CO_2_ experiments, and also the shape of the electron density being more suited to a linear triatomic molecule as opposed to a small, more spherical, diatomic molecule. In all cases, the CO_2_ molecules were soft restrained rather than modelled as rigid bodies. The positions of these soft restrained atoms were refined, followed by a final refinement of the soft restrained framework, resulting in excellent fits and satisfactory structural models for the entire pressure range. Individual occupancies and isotropic temperature factors of the atoms in each CO_2_ molecule were made equal, and the values for each molecule were refined simultaneously.

### 2.4. Preparation of the 25 wt% MFM-300(V^III^)/PIM-1 MMM

A solution casting method was employed for fabrication of the MFM-300(V^III^)/PIM-1 MMM. A quantity of 200 mg of MFM-300(V^III^) powder was pre-dispersed in 5 mL of CHCl_3_ with sonication and stirring followed by addition of 600 mg of PIM-1 (ref. 38) in 10 mL of CHCl_3_. In order to aid affinity between the MOF particles and polymer matrix, 20 μL of dimethoxysilylpropyl modified poly(ethyleneimine) was added to the casting solution as a surface modifier and the mixture stirred for 24 h. The mixture was evaporated to 5–7 mL and cast on a PTFE substrate and the solvent evaporated at room temperature. The resulting free-standing MMM was dried in an oven at 120 °C for 12 h prior to the permeation measurements.

### 2.5. Characterisation of the 25 wt% MFM-300(V^III^)/PIM-1 MMM

The purity of MFM-300(V^III^) was confirmed by powder X-ray diffraction before loading into the MMM. The membrane morphology was studied by scanning electron microscopy (SEM) using a Hitachi S-4800 Field-Emission Scanning Electron Microscope with Energy Dispersive X-ray (EDX) detector and cold cathode electron source. The membrane cross section was prepared *via* freeze fracturing using liquid N_2_. The sample was then coated with gold *via* sputtering using an Emitech coater. PXRD analysis was also carried out on a Philips X'Pert diffractometer to ensure retention of the crystallinity of the MOF once incorporated into the membrane.

### 2.6. Measurements of gas permeation of the 25 wt% MFM-300(V^III^)/PIM-1 MMM

Permeation was measured for pure gases (CO_2_, CH_4_, He, N_2_, H_2_) using the constant volume–variable pressure method.^39^ The recorded diffusion kinetics obeys Fick's first law of diffusion which relates the diffusive flux to the concentration under steady state. This postulates that a solute, in this case a gas, will move from an area of high concentration to an area of low concentration over a concentration gradient. This is described by the eqn (1),
1

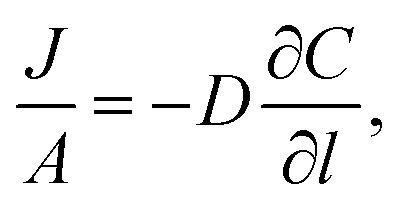

where *J* is the flux, *A* is the area, *D* is the diffusion coefficient, *C* is the concentration gradient and *l* is the thickness of the membrane. If we consider that the concentration gradient can be described by Henry's Law as eqn (2):

2 *C* = *S* × *p*

where *S* is the solubility coefficient and *p* is pressure, eqn (1) can be rewritten as eqn (3),
3

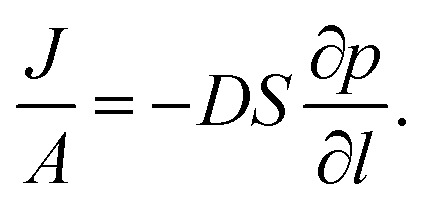




As permeation can be considered a solubility–diffusion process, the permeability coefficient *P* can be expressed as a product of the average diffusion coefficient, *D*, and solubility coefficient, *S* as shown in eqn (4):

4 *P* = *D* × *S*

The diffusion coefficient, *D*, is a measure of the mobility of the gas between the upstream and downstream side of the membrane and is determined by the packing and motion of the polymer segments and MOF particles and by the size and shape of the gas molecules. The solubility coefficient, *S*, is determined by the condensability of the penetrating gas molecules, by interactions with the polymer and MOF particles.

The perm-selectivity *α*_p_ of a given membrane for two gases (A and B) based upon single-component gas permeation measurements is given by eqn (5):

5 *α*_p_ = *P*_A_/*P*_B_

*α*_p_ represents for an ideal selectivity as it does not take into account interactions between the two gases or competitive processes. The same assumption was widely used to describe the gas adsorption selectivities, such as the ideal adsorbate solution theory (IAST).^40^ Similar equations can be used to obtain the diffusivity and solubility selectivities (*α*_d_ and *α*_s_) between two gases (A and B).

## 3. Results and discussion

### 3.1. Construction of the new remote control gas dosing system

The new remote control gas dosing system is now fully operational on Beamline I11 (Fig. 1b). It offers many advantages over the previous manual system,^26^ including:

• Complete programmability, leading to more efficient use of beam time;

• Much greater precision for gas loading, resulting in a higher reproducibility;

• Compatible with a number of toxic and corrosive gases;

• Safer operation, as users can operate it from outside of the X-ray hutch;

• Capability of mixing precisely up to 3 gases for sample loading.

Therefore, the development of this facility will enable new advanced crystallographic studies of crystalline porous materials to gain understanding of their function at the molecular level. A proof-of-concept study has been carried out on a V^III^-based MOF to determine the preferred binding domains for CO_2_ molecules in the pore in the presence and absence of N_2_ gas. The study has yielded important evidence for the observed high CO_2_/N_2_ adsorption selectivity.

### 3.2. Structural analysis of single-component gas loaded MFM-300(V^III^)

The complex MFM-300(V^III^) [V_2_(OH)_2_(L)] (H_4_L = biphenyl-3,3′,5,5′-tetracarboxylic acid) was synthesised by hydrothermal reaction of VCl_3_ and H_4_L in slightly acidic (HCl) water at 210 °C.^41^ MFM-300(V^III^), isolated as green microcrystalline powder, is isostructural to MFM-300(M) (M = Al, Ga, In),^3,19,22^ and comprises of chains of corner sharing [VO_4_(OH)_2_] octahedra, linked by mutually cis-μ_2_-OH groups, and further bridged by tetracarboxylate L^4–^ ligands. This arrangement generates 1D square-shape pore channels in a ‘wine rack’ array. Desolvated MFM-300(V^III^) displays a surface area of 1892 m^2^ g^–1^, a pore size of *ca.* 5.2 Å, and a total pore volume of 0.490 cm^3^ g^–1^ as determined from CO_2_ isotherm at 273 K, where the total uptake was recorded as 8.6 mmol g^–1^ at 1 bar. Desolvated MFM-300(V^III^) shows an exceptionally high CO_2_ adsorption capacity of 6.0 mmol g^–1^ (26.4 wt%) at 298 K and 1.0 bar, comparable to the best-behaving materials such as MOF-74(Mg) or CPO-27(Mg) (26–27.5 wt%) under same conditions.^42,43^ In contrast, desolvated MFM-300(V^III^) shows negligible N_2_ uptake, possibly due to the narrow pore window, leading to a high CO_2_/N_2_ selectivity of 81. We therefore sought to gain in-depth structural insights into the gas binding, and more importantly on competitive gas binding in this porous host.

**Fig. 2:**
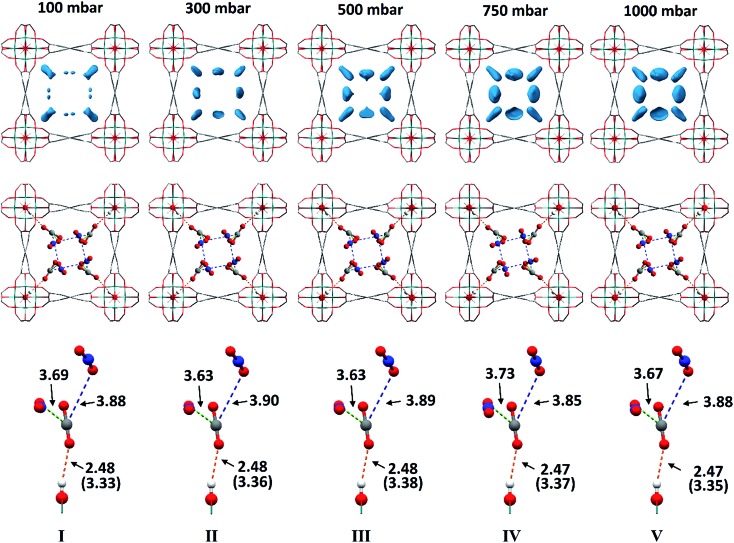
Top: Difference Fourier maps of CO_2_-loaded MFM-300(V^III^) from 100–1000 mbar viewed along the *c*-axis. Middle: Crystal structures of CO_2_-loaded MFM-300(V^III^) from 100–1000 mbar viewed along the *c*-axis. Bottom: Detailed view of the interaction of CO_2_-A with the V–OH group and the intermolecular dipole interactions with CO_2_-B. In the case of the OH ^A^O=C=O interaction, O ^A^O=C=O distances are shown in brackets. For clarity, only half of the positionally disordered CO_2_ molecules have been shown.

**Fig. 3:**
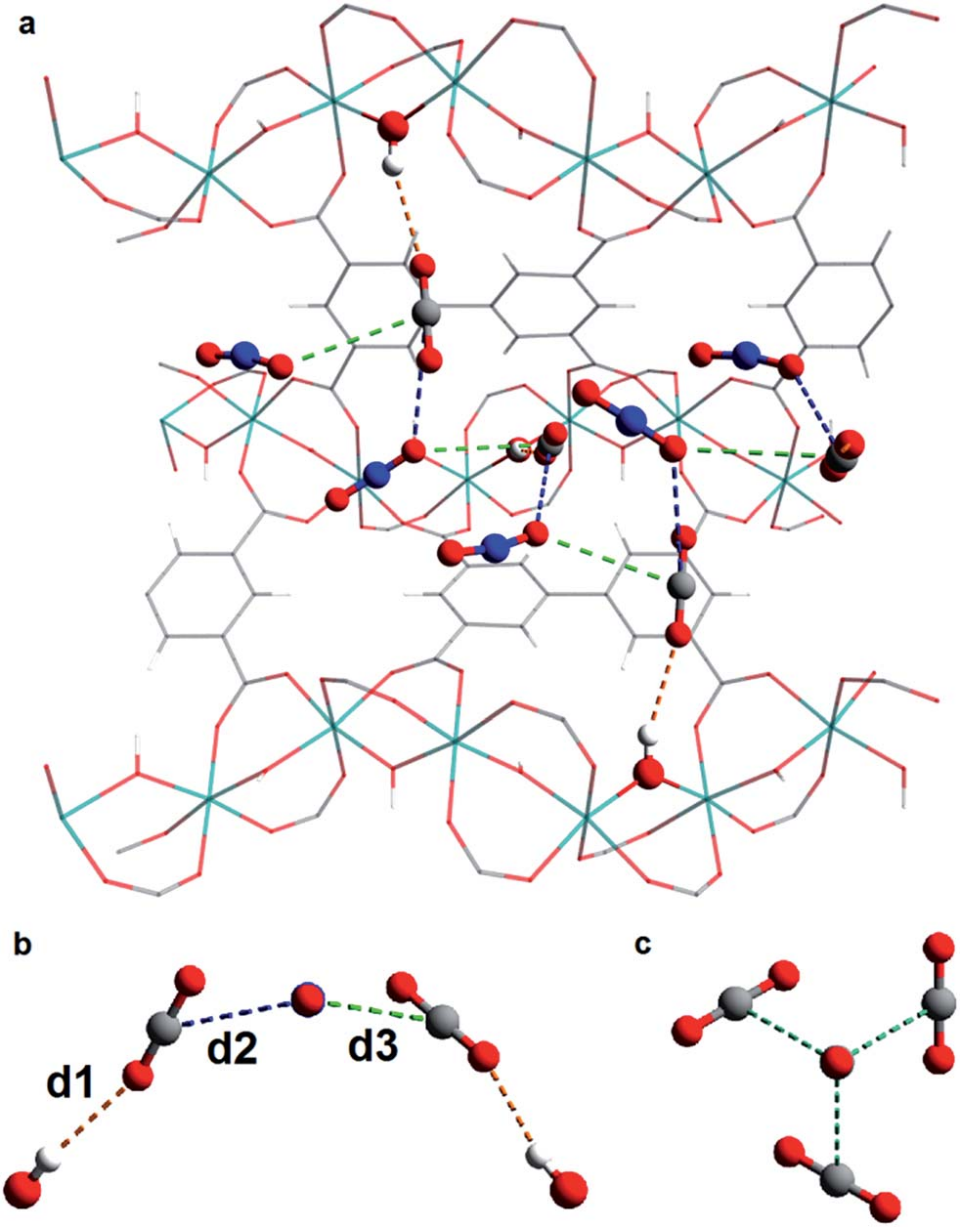
(a) View of structure of MFM-300(V^III^)·*x*CO_2_ along the *a* axis showing the infinite chiral network that propagates through the pore; (b) view of interactions between CO_2_-B and CO_2_-A in MFM-300(V^III^)·*x*CO_2_; (c) view of the structure of CO_2_ in the solid state at 150 K.

*In situ* PXRD data confirm the absence of structural phase change of MFM-300(V^III^) as a function of CO_2_ loading. Analysis of the bare MOF indicates the complete removal of solvent molecules from the pore. Difference Fourier analysis of the CO_2_-loaded structures revealed two well-defined regions of residual electron density within the pore of MFM-300(V^III^): one situated above the hydroxyl group of the framework and the other located adjacent to this region (Fig. 2). Soft-restrained CO_2_ molecules were added to the structural model of bare MFM-300(V^III^), and their positions, orientations and occupancies refined, revealing two distinct binding sites (A and B) across the entire pressure range. It is important to note that for clarity, half of the positionally disordered CO_2_ molecules are not shown in Fig. 2. CO_2_-A forms a hydrogen bond interaction with the μ_2_-OH group of the framework. CO_2_-B interacts with CO_2_-A *via* two separate dipole–dipole interactions resulting in distorted T-shape arrangement. When comparing these interactions with those found in dry ice at 150 K (ref. 44) (Fig. 3), there are many similarities, the most striking of which is the manner in which CO_2_-B ‘sandwiches’ itself between two molecules of CO_2_-A, maximising the number of dipole–dipole interactions between the molecules. The C=O^A^ <svg xmlns="http://www.w3.org/2000/svg" version="1.0" width="16.000000pt" height="16.000000pt" viewBox="0 0 16.000000 16.000000" preserveAspectRatio="xMidYMid meet"><metadata> Created by potrace 1.16, written by Peter Selinger 2001-2019 </metadata><g transform="translate(1.000000,15.000000) scale(0.005147,-0.005147)" fill="currentColor" stroke="none"><path d="M0 1440 l0 -80 360 0 360 0 0 80 0 80 -360 0 -360 0 0 -80z M1040 1440 l0 -80 360 0 360 0 0 80 0 80 -360 0 -360 0 0 -80z M2080 1440 l0 -80 320 0 320 0 0 80 0 80 -320 0 -320 0 0 -80z"/></g></svg> C^B^ angle of interaction across the entire pressure range (128–139°) is also similar to C=O′C′′ angle (132.35°) in solid CO_2_. The intermolecular distances for solid CO_2_, however, are shorter than those observed in MFM-300(V^III^), with a O=C=OC(=O)_2_ distance of 3.11 Å, compared to distances from 3.63(6)–3.89(4) Å found in MFM-300(V^III^). This is most likely due to the solid state structure of CO_2_ being obtained at a much lower temperature than of CO_2_-loaded MFM-300(V^III^). The combination of these interactions forms an infinite chiral network of CO_2_-A and CO_2_-B that propagates along the pore (Fig. 3). These structures vary significantly from that of MFM-300(Al)·3.2CO_2_ studied by PXRD at 273 K.^3^ In MFM-300(Al) the two CO_2_ sites align to create discrete T-shaped O=C=OC(=O)_2_ units above each hydroxyl. These differences can be explained by the slightly narrower pore of MFM-300(Al) having more of a confinement effect than MFM-300(V^III^) on the CO_2_ molecules.

**Fig. 4:**
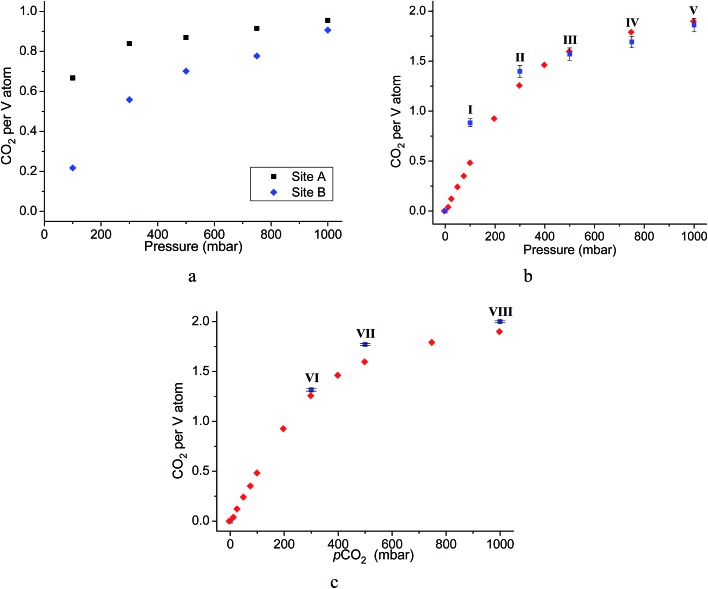
(a) Plot of CO_2_ occupancies from single component CO_2_ adsorption experiments as a function of pressure (normalised to the site occupancy of the V center); (b) plot of combined CO_2_ occupancies from single component CO_2_ adsorption experiment as a function of pressure (blue squares) overlaid with the gravimetric CO_2_ isotherm of MFM-300(V^III^) (red diamonds); (c) plot of combined CO_2_ occupancies from dual component N_2_/CO_2_ adsorption experiment as a function of pressure (blue squares) overlaid on gravimetric CO_2_ isotherm of MFM-300(V^III^) (red diamonds).

Throughout the 5 different loadings of CO_2_ [100 (I), 300 (II), 500 (III), 750 (IV) and 1000 (V) mbar], the position and orientation of CO_2_-A relative to the oxygen atom of the hydroxyl moiety remain consistent with the orientation of CO_2_-B changing only slightly over the pressure range. The distances for these interactions are summarised in Table 1. Inspection of the crystallographic occupancies of each site with respect to pressure (Fig. 2) shows that each site populates concurrently, with site A having a higher occupancy than site B at each pressure. The difference in site occupancy is more pronounced at low pressure where host–guest interaction is dominating the uptake. With increased pressure, the difference on site occupancy decreases. This suggests the presence of a coherent cooperativity between two sites, and implies a dependency of site B on the presence of site A. Combined occupancies of the two sites of CO_2_ atoms from these refinements were plotted as a function of pressure and compared to the CO_2_ adsorption isotherm of MFM-300(V^III^) at 273 K (Fig. 4). The occupancy plot shows an excellent agreement with the isotherm, further validating the accuracy of the structural models, whilst also confirming the high accuracy of the remote control gas dosing system. Thus, these results highlight the dynamic nature of the adsorbed gases within the pore as the pressure changes, and suggest that the lowest energy crystallographic position for the guest molecules is affected by their crystallographic occupancy.

**Table 1:** Distances in the structures MFM-300(V^III^)·*x*CO_2_ obtained from single and dual component experiments. Distance *d*_1_ is the distance from O atom of CO_2_-A to O atom of the framework hydroxy group. Distances *d*_2_ and *d*_3_ are distances between C atom of CO_2_-A and O atoms of CO_2_-B, respectively. These interactions are highlighted in Fig. 2, 3 and 5

Structure	CO_2_ pressure (mbar)	*d*_1_ (Å)	*d*_2_ (Å)	*d*_3_ (Å)
I	100	3.33(1)	3.69(7)	3.88(6)
II	300	3.36(1)	3.63(6)	3.90(5)
III	500	3.38(1)	3.63(6)	3.89(5)
IV	750	3.37(1)	3.73(4)	3.85(4)
V	1000	3.35(1)	3.67(4)	3.88(4)
VI	300^*a*^	3.35(2)	3.60(2)	3.99(2)
VII	500^*a*^	3.34(2)	3.73(4)	3.90(4)
VIII	1000^*a*^	3.37(1)	3.72(3)	3.93(3)

^*a*^Indicates the partial pressure of CO_2_ in the equimolar CO_2_/N_2_ mixture.

Similar difference Fourier analysis of N_2_-loaded MFM-300(V^III^) revealed no specific regions of residual electron density in the pore, and refinement of the empty framework against the N_2_-loaded data revealed no apparent contribution from N_2_ molecules to the structure, consistent with the ultra-low N_2_ uptake in the corresponding isotherm.

### 3.3. Structural analysis of dual-component loading of an equimolar mixture of CO_2_/N_2_ in MFM-300(V^III^)

**Fig. 5:**
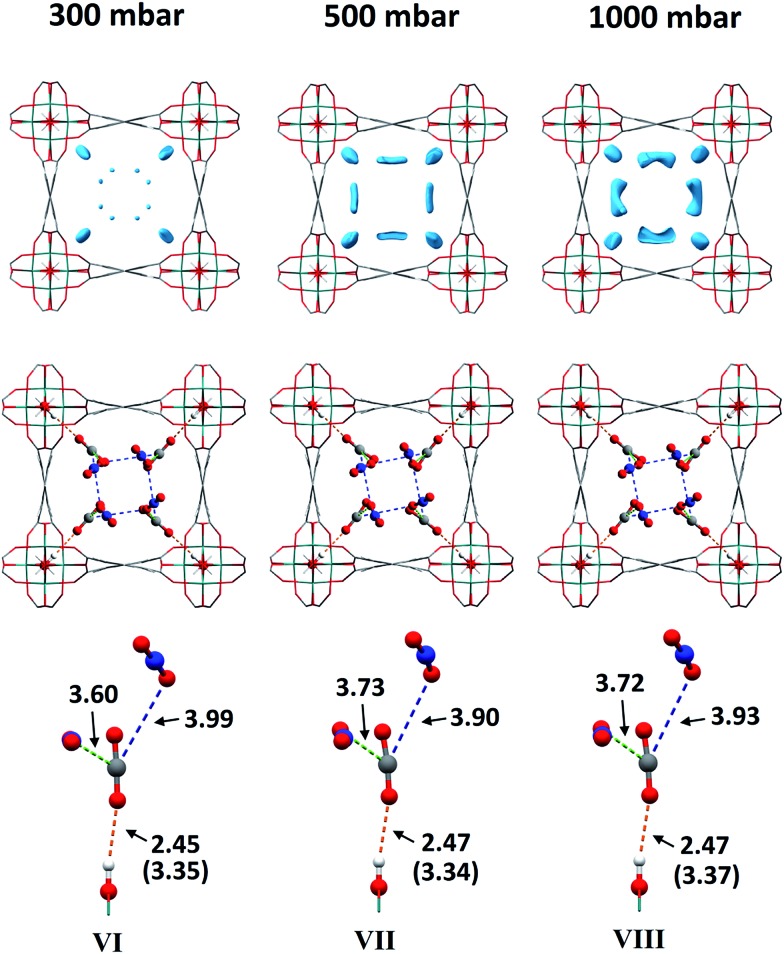
Top: Difference Fourier maps of CO_2_/N_2_-loaded MFM-300(V^III^) from 300–1000 mbar viewed along the *c*-axis. Middle: Structures of CO_2_/N_2_-loaded MFM-300(V) from dual component experiments from 300–1000 mbar viewed along the *c*-axis. Bottom: View of the interactions of CO_2_-A with the framework hydroxyl group and its two interactions with CO_2_-B. In the case of the OH ^A^O=C=O interaction, O ^A^O=C=O distances are shown in brackets. For clarity, only half of the positionally disordered CO_2_ molecules are shown.

As for the above structural refinements, difference Fourier analysis revealed two regions of high electron density and are attributed to CO_2_ molecules due to the similarity in position and shape to those in the single component CO_2_ refinements. The electron density was more elongated than spherical, suggesting the presence of the linear, triatomic, CO_2_ as opposed to the more spherical N_2_. The refinement of two soft-restrained CO_2_ molecules in these positions confirmed the presence of the same two binding sites (A and B) found in the single component CO_2_ structures (Fig. 5). No adsorption sites could be found for N_2_, most likely due to the poor uptake of N_2_ at 273 K in this material. Again, the positions of CO_2_-A and CO_2_-B across three loadings (VI, VII, VIII) remain consistent with those found in the single component CO_2_ structures. The CO_2_ occupancy plot from these refinements (Fig. 4c) again show excellent agreement with both the adsorption isotherm and the occupancy plot from the single component experiment, which shows that CO_2_ binding in MFM-300(V^III^) does not change significantly in the presence of N_2_. Thus, this PXRD experiment on mixed gas loading affords direct structural insight into the competitive gas binding, underpinning the high adsorption selectivity of this MOF.

### 3.4. Analysis of gas permeation in MFM-300(V^III^)/PIM-1 under dynamic conditions

**Fig. 6:**
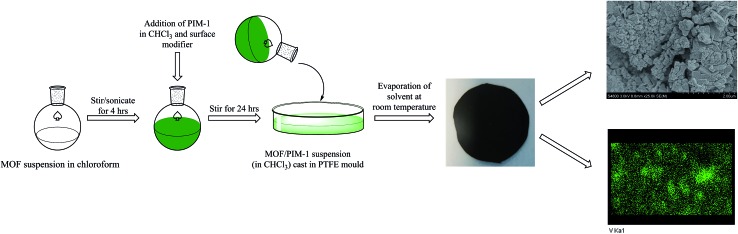
Schematic representation of the preparation of the MMM derives from MFM-300(V^III^) and PIM-1 with a photograph of the fabricated membrane and views by SEM and EDX.

**Fig. 7:**
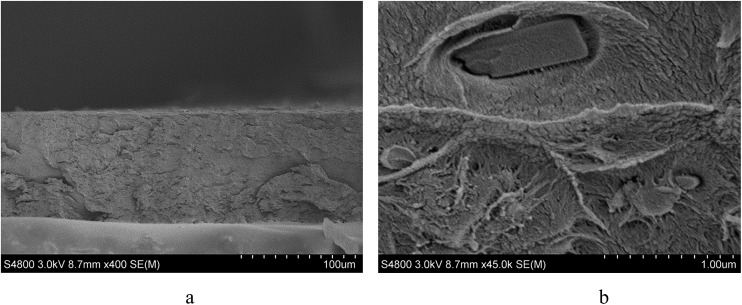
SEM images obtained using a Hitachi S-4800 Field-Emission Scanning Electron Microscope at 3 kV of (a) cross-section of the fabricated MMM at 100 μm scale; (b) image of a single particle showing good affinity between the polymer matrix and the MOF particle.

**Fig. 8:**
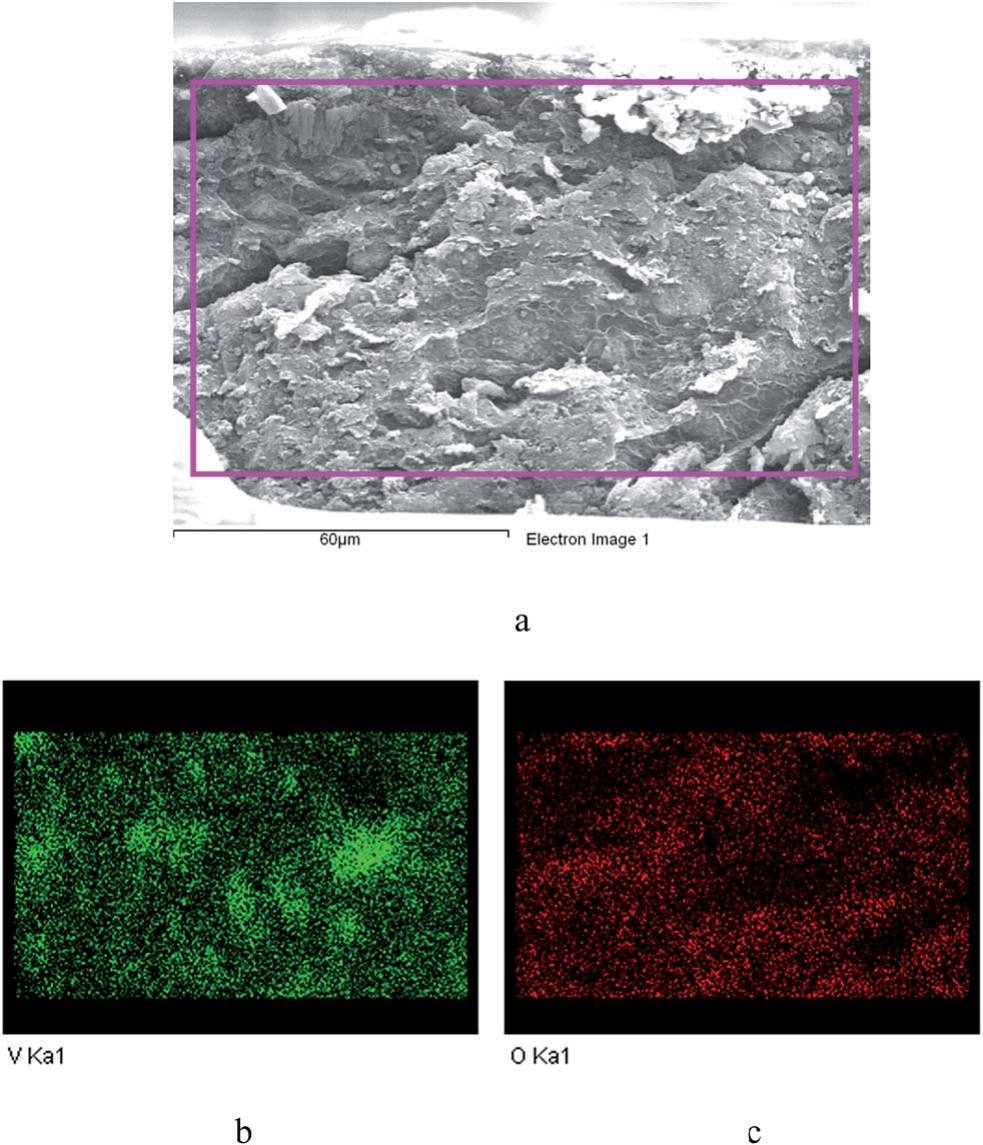
EDX elemental maps showing a uniform distribution of the MFM-300(V^III^) through the PIM-1 membrane. (a) SEM image of a cross-section of the membrane; (b) cross section elemental map of V in the membrane; (c) cross section elemental map of O in the membrane.

**Fig. 9:**
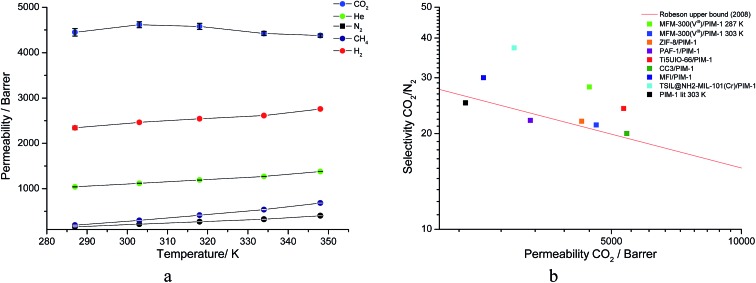
(a) Plot of change in gas permeability as a function of temperature. (b) Plot of the position of MFM-300(V^III^)/PIM-1 at 287 K and 303 K in comparison to bare PIM-1 at 303 K (ref. 38) and other PIM-1 based MMMs for CO_2_/N_2_ selectivity.

The membrane was cast from solution with a surface modifier (Fig. 6). This allows fabrication of a uniform membrane with a homogenous distribution of MOF particles, and more significantly, without any macro-voids between the MOF particles and the polymer matrix as indicated by SEM analysis (Fig. 7). EDX elemental analysis also confirms the even distribution of the MOF throughout the membrane (Fig. 8). PIM-1 was used for this study due to its proven gas separation performance and inherent porosity.^38^ Single gas (CO_2_, N_2_, CH_4_, H_2_ and He) tests were performed on a 25 wt% MFM-300(V^III^) loaded MMM in order to assess permeation performance (Table S3†). Addition of MFM-300(V^III^) showed an improvement in CO_2_ permeation and ideal CO_2_/N_2_ selectivity over that of the bare PIM-1. The improvement in CO_2_ permeation in the MMM can be primarily attributed to the high chemical affinity shown of the 1D pore channels of MFM-300(V^III^) and CO_2_ molecules as can be seen from the *in situ* PXRD studies described above. All permeation measurements were carried out at a range of temperatures (287–343 K) and a general increase in permeation was observed with rising temperature as expected, with the exception of CO_2_ which showed an initial increase in permeability between 287 K and 303 K followed by a decrease in permeability (Fig. 9). The initial increase in CO_2_ permeability between 287 K and 303 K, from 4450 Barrer to 4617 Barrer, could be explained by the increased speed of diffusion of the gas through the membrane, from 76 × 10^8^ cm^2^ s^–1^ to 107 × 10^8^ cm^2^ s^–1^ and although solubility decreases between these temperatures this does not offset the observed diffusion. The subsequent decrease in permeability seen at increasing temperatures can be explained by the decreased solubility of CO_2_ with the diffusion not significantly increasing to offset this due to the strong interactions between the CO_2_ molecules and the hydroxy groups present within the pores of MFM-300(V^III^). This is further supported by the permeability trend shown with increasing temperature for other gases tested which have a much reduced interaction with the MOF host.

On the Robeson plot, it is evident that this MMM improves on the performance of the bare polymer PIM-1 and falls above the 2008 Robeson upper bound in terms of the ideal selectivity and CO_2_ permeability (Fig. 9). Moreover, MFM-300(V^III^)/PIM-1 performs very competitively to other reported best-behaving PIM-1 based MMMs (Table 2). The new MMM also performs well when compared to membranes which have used other polymer supports. An example of this is a UiO-66-NH_2_/PEBA membrane fabricated by Shen *et al.* ^51^ which showed a higher CO_2_/N_2_ selectivity of 66 but a much lower CO_2_ permeability of 87 Barrer compared to over 4000 Barrer for MFM-300(V^III^)/PIM-1. Therefore, the dynamic experiments demonstrate and validate the potential of utilising this MFM-300(V^III^) in CO_2_ separation, and the data may also be improved by applying nanosized MOF to increase the MOF surface per volume element and the interaction of MOF with the host polymer.

**Table 2:** Comparison of the permeability and selectivity for MMM from this work and other PIM-1-based MMMs from the literature

Filler	Loading (wt%)	*P*_CO_2__/Barrer	*α* (CO_2_/N_2_)	Operational conditions	Reference
MFM-300(V^III^)	25	4450	28.04	287 K, 1 bar	This work
MFM-300(V^III^)	25	4617	21.28	303 K, 1 bar	This work
ZIF-8	39	4270	21.89	295 K, 1 bar	45
PAF-1	30	3250	22	298 K, 1 bar	46
Ti5UIO-66	5	5340	24	298 K, 2 bar	47
CC3	30	5430	20	298 K, 1 bar	48
MFI	23.5^*a*^	2530	30	298 K, 1 bar	49
TSIL@NH_2_-MIL-101(Cr)	5	2979	37.24	298 K, 3 bar	50

^*a*^Volumetric loading for this sample as v%.

## 4. Conclusions

In summary, a new remote control gas dosing system capable of millibar precision and fully automated operation has been successfully implemented on the powder diffraction Beamline I11 at Diamond Light Source. This system has shown unique *in situ* structural characterisation of MFM-300(V^III^) as a function of loading of CO_2_, N_2_ and an equimolar mixture of CO_2_/N_2_. Rietveld structural analysis confirmed the presence of two distinct CO_2_ sites within the MOF pore with a hydrogen bond interaction between the framework μ-OH and CO_2_-A, and two further intermolecular dipole interactions between CO_2_-B and CO_2_-A. The positions of CO_2_-A and CO_2_-B were found to remain generally consistent across the entire pressure range. Upon dosing with an equimolar mixture of CO_2_ and N_2_, a similar network of CO_2_-A and CO_2_-B was observed in MFM-300(V^III^). Occupancy plots from both the single and dual component experiments show excellent agreement with the adsorption isotherms, confirming the precision of the gas dosing system and the structural analysis. The fabrication of a MMM based upon PIM-1 doped with 25 wt% MFM-300(V^III^) has been demonstrated. The inclusion of the MOF leads to an increased permeation to CO_2_ and an improvement of the ideal gas separation performance for CO_2_/N_2_ over the bare polymer. This increased CO_2_ permeation is likely due to the high chemical affinity of MFM-300(V^III^) for CO_2_ as confirmed by *in situ* PXRD studies. Importantly, the newly formed MMM also falls above the Robeson 2008 upper bound for membrane performance at both 287 K and 303 K, demonstrating the potential of utilising this material in the application of CCS owing to its optimal compromise between selectivity and permeability.
