# Supplementary material for: Stepwise observation and quantification and mixed matrix membrane separation of CO2 within a hydroxy-decorated porous host
Source: Chem Sci. 2017 Feb 27;8(4):3239–48. doi: 10.1039/c6sc04343g (PMC5414597; doi:10.1039/c6sc04343g)
Supplement: Supplementary file 1 [file SC-008-C6SC04343G-s001.pdf]

Electronic Supplementary Material (ESI) for Chemical Science.
This journal is © The Royal Society of Chemistry 2017

# Electronic Supplementary Information

## Stepwise Observation and Quantification and Mixed Matrix Membrane Separation of $CO_2$ within a Hydroxyl-Decorated Porous Host

Christopher G. Morris,[1,2] Nicholas M. Jacques,[1] Harry G. W. Godfrey,[1] Tamoghna Mitra,[3] Detlev Fritsch,[4] Zhenzhong Lu,[1] Claire A. Murray,[2] Jonathan Potter,[2] Tom M. Cobb,[2] Fajin Yuan,[2] Chiu C. Tang[2]*, Sihai Yang[1]* and Martin Schröder[1]*

1. School of Chemistry, University of Manchester, Oxford Road, Manchester, M13 9PL, UK.
Sihai.Yang@manchester.ac.uk; M.Schroder@manchester.ac.uk
2. Diamond Light Source, Harwell Science and Innovation Campus, Didcot, Oxfordshire, OX11 0DE, UK.
chiu.tang@diamond.ac.uk
3. Department of Chemistry, University of Liverpool, Liverpool, L69 7ZD, UK.
4. Fraunhofer IAP, FB3, Geiselbergstrasse 69, Potsdam-Golm, 14476, Germany.

**ESI Index**

**1. Gas Panel**

**2. Unit Cell Analysis**

**3. Rietveld Refinement Details**

**4. Calculation of Adsorption Selectivity of $CO_2/N_2$**

**5. Atomic Coordinates**

**6. Synthesis of PIM-1**

**7. PXRD of mixed matrix membrane MFM-300(VIII)/PIM-1**

**8. SEM of mixed matrix membrane MFM-300(VIII)/PIM-1**

**9. Results of Permeation experiments**

## 1. Gas Panel

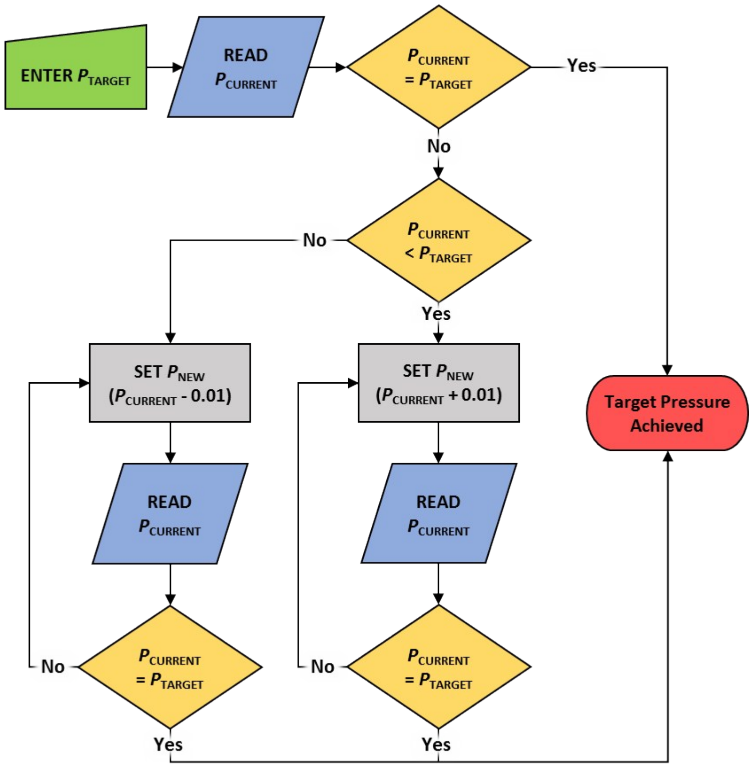


**Figure S1.** Flow diagram of the logic used for sample dosing. The loops ensure control of gradual incremental pressure.

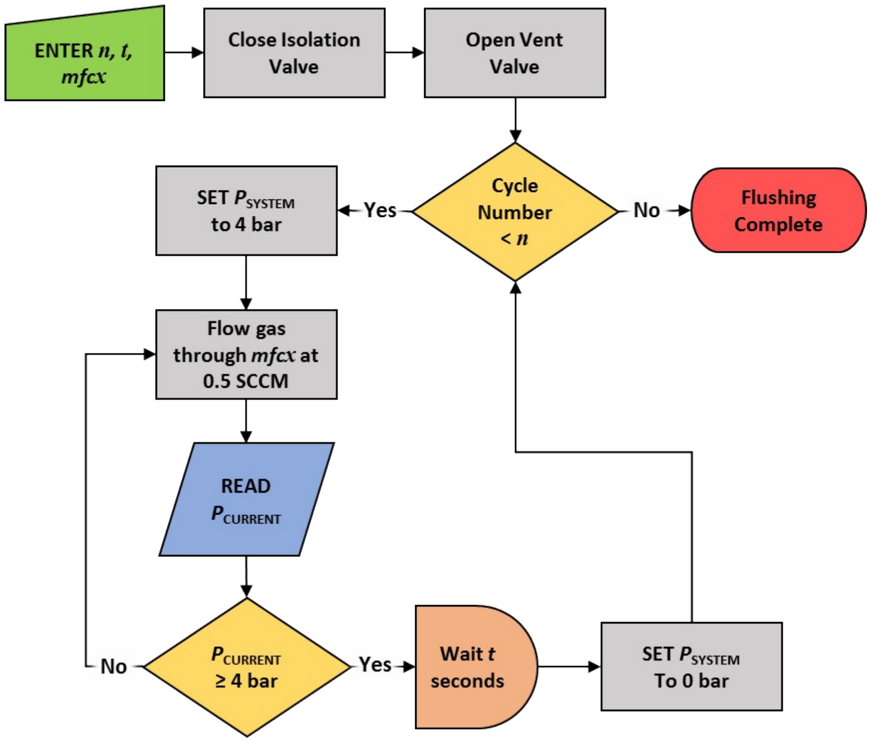


**Figure S2.** Flow diagram of the logic used for flushing the system.

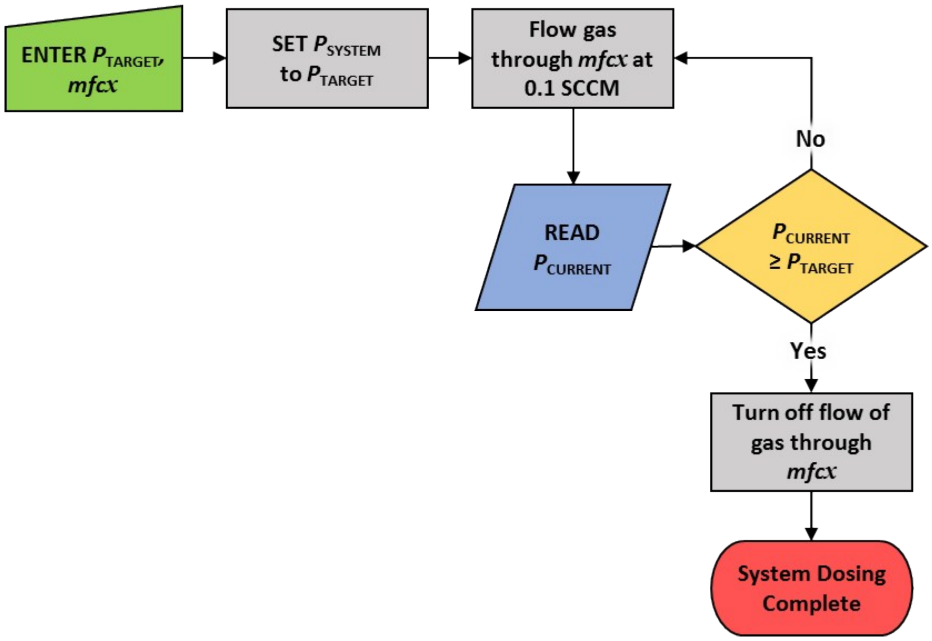


**Figure S3.** Flow diagram of the logic used for dosing the system.

## 2. Unit Cell Analysis

Plots of *a* and *c* parameters derived from the Pawley refinements of the single component $CO_2$ experiments as a function of pressure are shown in Figure S4. A general decrease in length is observed for each parameter. From the structural refinements, this trend can be explained by the $\mu$-OH---O=C=O and the two O=C=O---C(=O)$_2$ interactions forming a network of interactions that generate an increase in 'attractive' force within the pore, and this causes the framework to contract along both axes. Conversely, lattice parameters obtained from the dual component experiments show a general increase in length of the *a* and *c* parameters with increasing pressure (Figure S5), likely due to the presence of disordered non-interacting $N_2$ inside the pore causing the channel to expand as a result of repulsion. This is supported by the fact that expansion along the *a* axis, an axis very much dependent on the pore width, is much greater than expansion along the *c* axis.

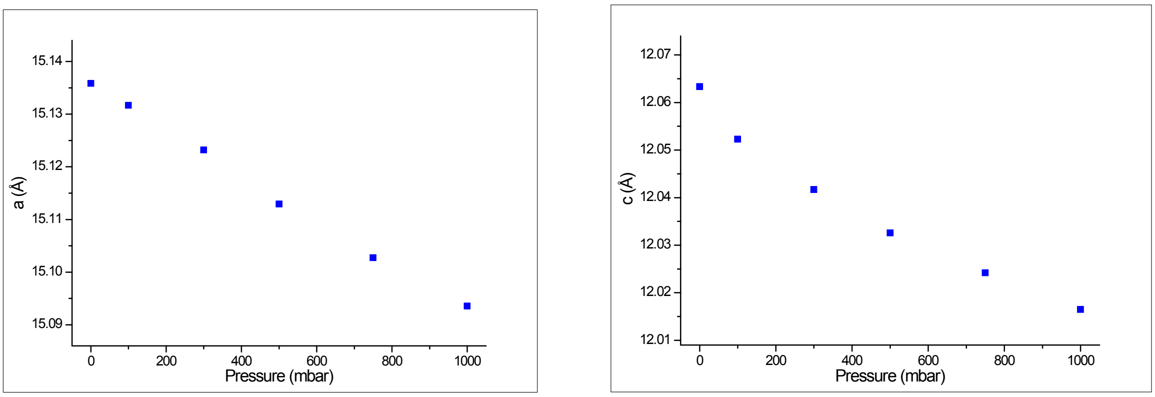


**Figure S4.** Left: Plot of parameter *a* vs. pressure from the single component $CO_2$ adsorption experiment. Right: Plot of parameter *c* vs. pressure from single component $CO_2$ adsorption experiment.

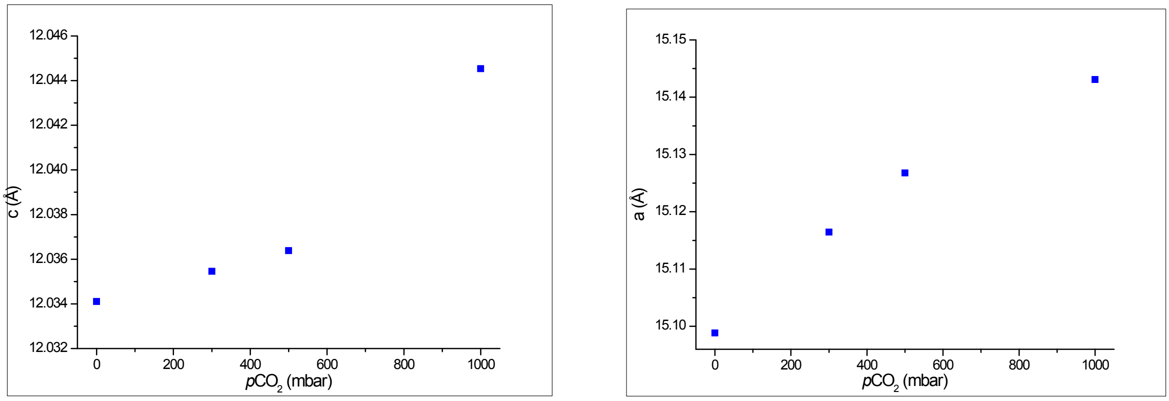


**Figure S5**. Left. Plot of parameter *a* vs. pressure from dual component $N_2/CO_2$ adsorption experiment. Right: Plot of *c* parameter vs. pressure from dual component $N_2/CO_2$ adsorption experiment.

**3. Rietveld refinement details**

| Pressure (mbar) | *a* (Å) | *c* (Å) | Volume ($Å^3$) | $R_{wp}$ | GOF |
|---|---|---|---|---|---|
| 0 | 15.13549(4) | 12.06324(4) | 2763.48(2) | 9.538 | 2.550 |
| 100 | 15.13178(3) | 12.05232(3) | 2759.63(1) | 6.850 | 1.803 |
| 300 | 15.12359(4) | 12.04198(3) | 2754.28(2) | 6.684 | 1.758 |
| 500 | 15.11334(4) | 12.03274(4) | 2748.43(2) | 6.613 | 1.735 |
| 750 | 15.10345(4) | 12.02468(4) | 2743.00(2) | 6.392 | 1.677 |
| 1000 | 15.09548(5) | 12.01748(5) | 2738.47(2) | 6.647 | 1.745 |

| *pp*$CO_2$ (mbar) | *a* (Å) | *c* (Å) | Volume ($Å^3$) | $R_{wp}$ | GOF |
|---|---|---|---|---|---|
| 300 | 15.116456(86) | 12.0355(1) | 2750.190(4) | 7.369 | 2.070 |
| 500 | 15.126742(89) | 12.0363(1) | 2754.12(4) | 7.602 | 2.176 |
| 1000 | 15.142282(62) | 12.04491(6) | 2761.76(3) | 9.031 | 2.592 |

**Table S1.** Unit cell and Rietveld refinement results from single component $CO_2$ adsorption experiment

**Table S2.** Unit cell and Rietveld refinement results from equimolar $CO_2/N_2$ adsorption experiment

**Fits of Rietveld refinements from single component $CO_2$ adsorption experiments**

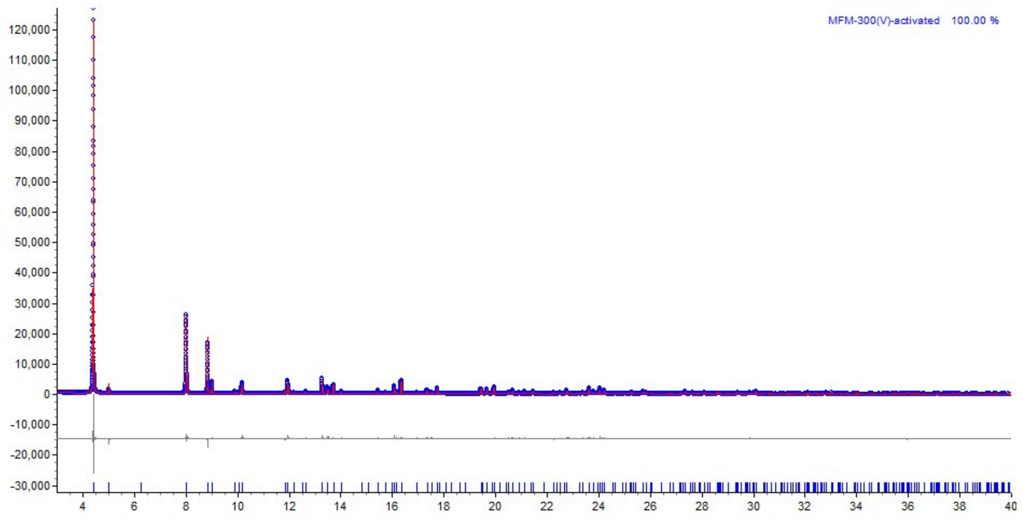


**Figure S6**. Rietveld refinement plot of MFM-300($V^{III}$). Blue open circle = observed; red line = calculated; grey line = difference.

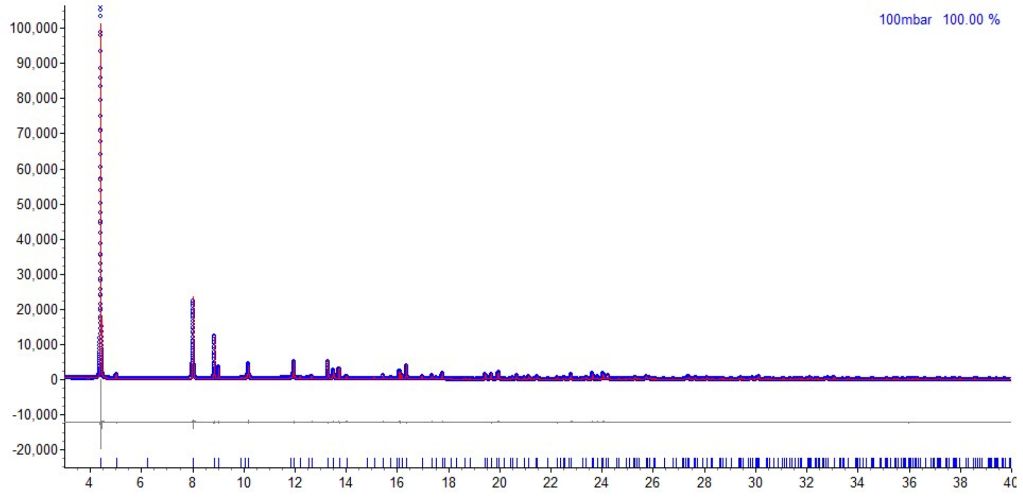


**Figure S7**. Rietveld refinement plot of MFM-300($V^{III}$)-I. Blue open circle = observed; red line = calculated; grey line = difference.

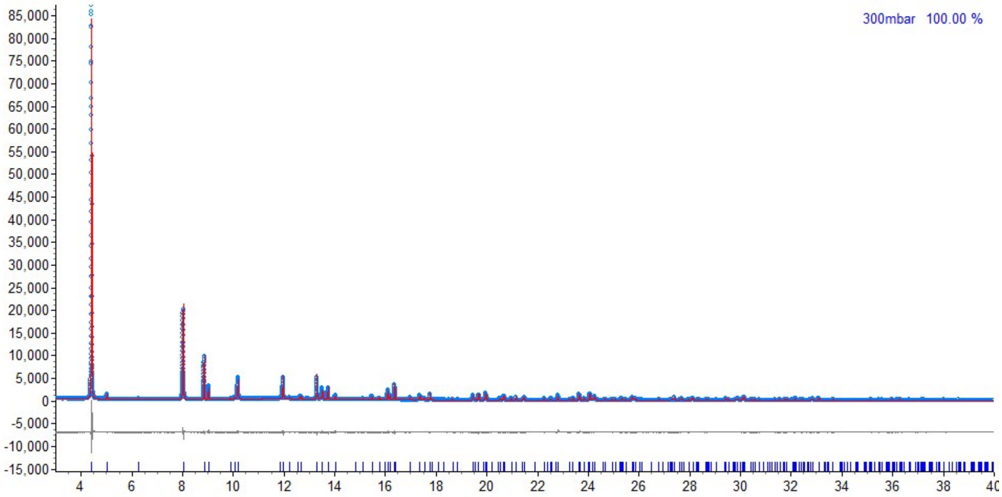


**Figure S8**. Rietveld refinement plot of MFM-300($V^{III}$)-II. Blue open circle = observed; red line = calculated; grey line = difference.

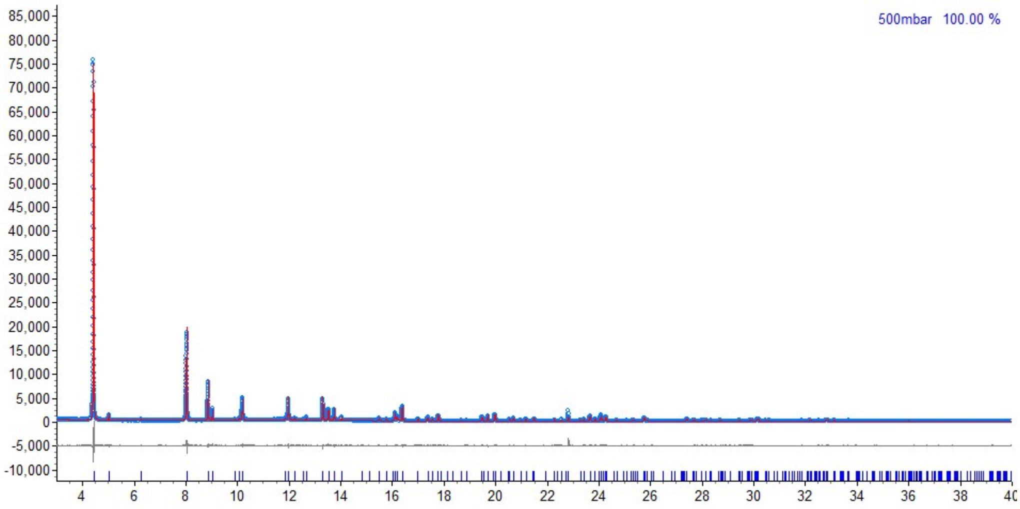


**Figure S9**. Rietveld refinement plot of MFM-300($V^{III}$)-III. Blue open circle = observed; red line = calculated; grey line = difference.

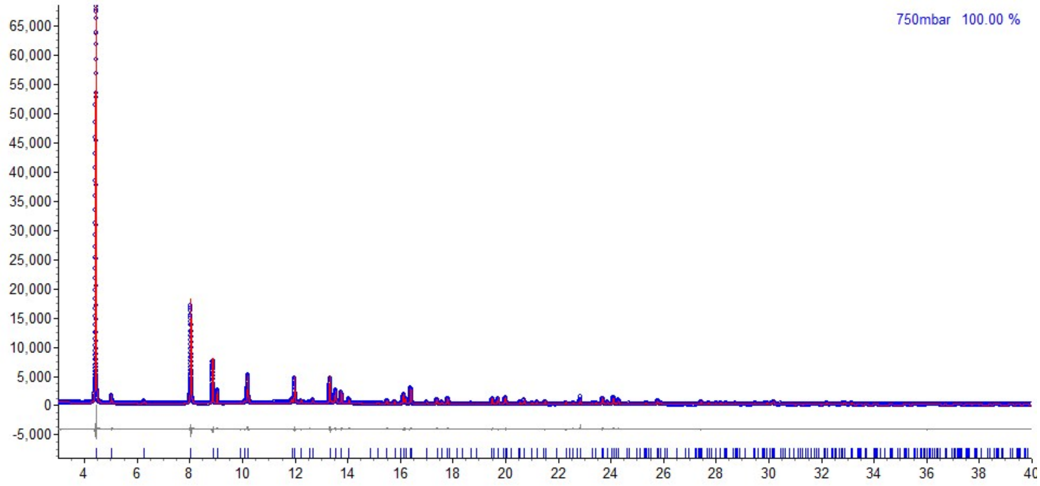


**Figure S10**. Rietveld refinement plot of MFM-300($V^{III}$)-IV. Blue open circle = observed; red line = calculated; grey line = difference.

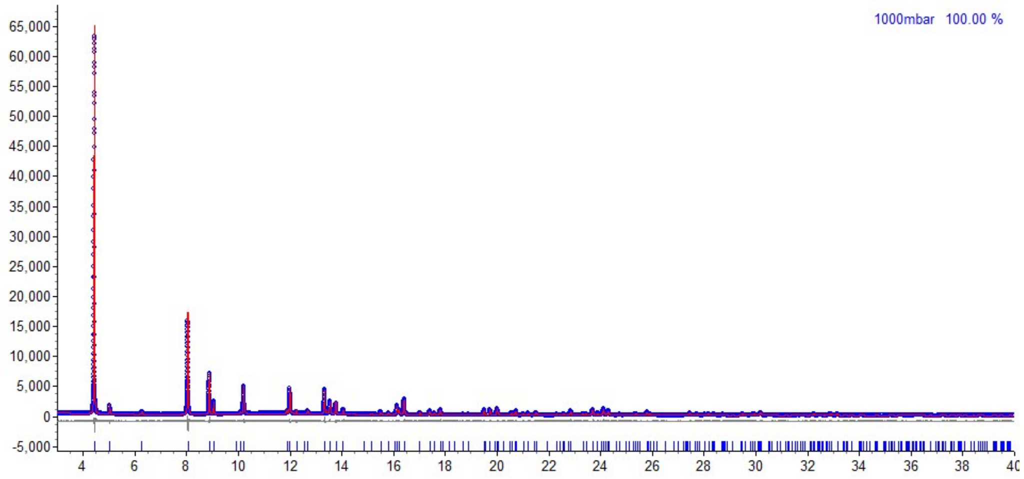


**Figure S11**. Rietveld refinement plot of MFM-300($V^{III}$)-V. Blue open circle = observed; red line = calculated; grey line = difference.

**Fits of Rietveld refinements from dual component $N_2/CO_2$ adsorption experiment**

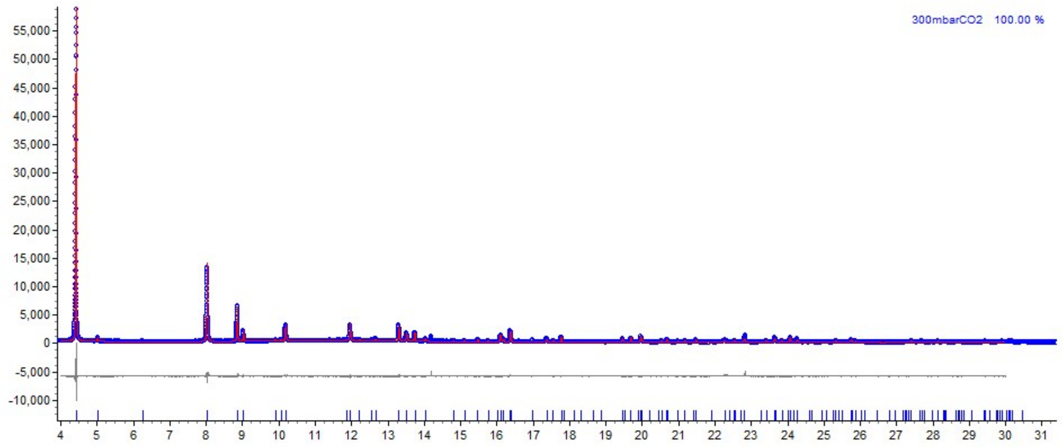


**Figure S12**. Rietveld refinement plot of MFM-300($V^{III}$)-VI. Blue open circle = observed; red line = calculated; grey line = difference.

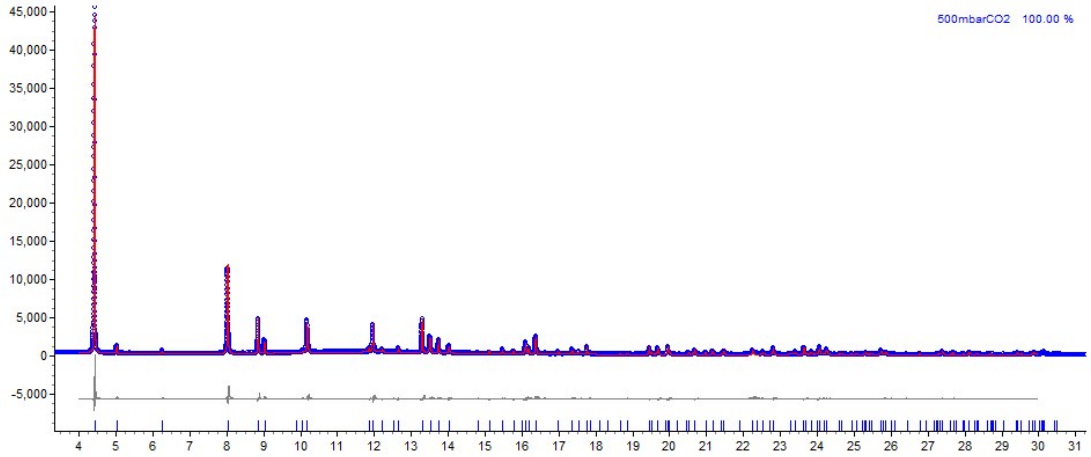


**Figure S13**. Rietveld refinement plot of MFM-300($V^{III}$)-VII. Blue open circle = observed; red line = calculated; grey line = difference.

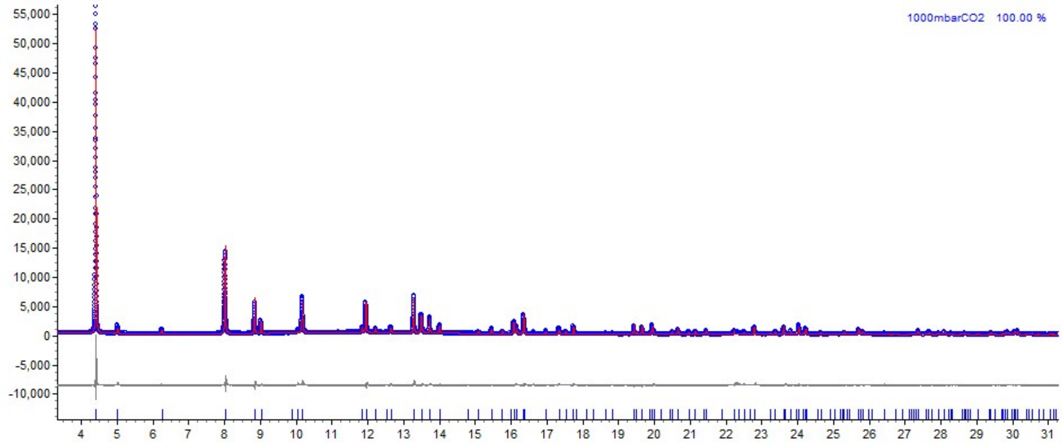


**Figure S14**. Rietveld refinement plot of MFM-300($V^{III}$)-VII. Blue open circle = observed; red line = calculated; grey line = difference.

## 4. Calculation of Adsorption Selectivity of $CO_2/N_2$

The $CO_2$ and $N_2$ sorption data for MFM-300($V^{III}$) measured at 273 K were fitted by the virial equation (1).

$$\ln(P) = \ln(Va)+(a0+a1*Va+a2*Va^2 \ldots+ a6*Va^6)/T+(b0+b1*Va) \quad (1)$$

where ***P*** is pressure, ***Va*** is amount adsorbed, ***T*** is temperature, and **a0**, **a1**, **a2** … ,**a6** and **b0**, **b1** are temperature independent empirical parameters.

Henry's constant (***KH***) is calculated from equation (2), where ***T*** is temperature.

$$KH = \exp(-b0)\cdot\exp(-a0/T) \quad (2)$$

The Henry's Law selectivity ($S_{ij}$) for gas *i* over *j* at 273K and 298 K is calculated from the following equation (3).

$$S_{ij}= K_{Hi}/K_{Hj} \quad (3)$$

Virial fitting results and Henry's constants $K_H$ for $CO_2$ and $N_2$ in MFM-300($V^{III}$) from isotherm data at 273 K

| | $CO_2$ | $N_2$ |
|---|---|---|
| *KH* (mol $g^{-1}$ $bar^{-1}$) | 27.5 | 0.339 |
| Fitting $R^2$ | >0.999 | 0.993 |
| $CO_2/N_2$ Selectivity | 81 | |

## 5. Atomic Coordinates

Atomic Coordinates for activated MFM-300($V^{III}$)

| Site | *x* | *y* | *z* | Occupancy | $B_{iso}$ (Å$^2$) |
|---|---|---|---|---|---|
| V | 0.68986(7) | 0.31014(7) | 0.5 | 1 | 1.00(3) |
| O1 | 0.7443(3) | 0.25 | 0.625 | 1 | 1.01(5) |
| O2 | 0.89717(19) | 0.29233(19) | 0.9829(2) | 1 | 1.01(5) |
| O3 | 0.6189(2) | 0.3729(2) | 0.3826(2) | 1 | 1.01(5) |
| C1 | 0.5919(3) | 0.3589(3) | 0.7108(4) | 1 | 1.63(7) |
| C2 | 0.5414(3) | 0.4319(3) | 0.7632(4) | 1 | 1.63(7) |
| C3 | 0.5 | 0.5 | 0.7080(5) | 1 | 1.63(7) |
| C4 | 0.5456(3) | 0.4327(3) | 0.8818(3) | 1 | 1.63(7) |
| C5 | 0.5 | 0.5 | 0.9372(4) | 1 | 1.63(7) |
| H3 | 0.5 | 0.5 | 0.631(3) | 1 | 1.96(8) |
| H4 | 0.57318 | 0.38162 | 0.90735 | 1 | 1.96(8) |
| H1 | 0.806 | 0.25 | 0.625 | 1 | 1.96(8) |

Atomic Coordinates for MFM-300(V)-100mbar$CO_2$

| Site | *x* | *y* | *z* | Occupancy | $B_{iso}$ (Å$^2$) |
|---|---|---|---|---|---|
| V | 0.690570(50) | 0.309430(50) | 0.5 | 1 | 3.749(24) |
| O1 | 0.74830(21) | 0.25 | 0.625 | 1 | 3.704(39) |
| O2 | 0.89895(15) | 0.28981(14) | 0.99186(21) | 1 | 3.704(39) |
| O3 | 0.62028(16) | 0.38135(16) | 0.39090(17) | 1 | 3.704(39) |
| C1 | 0.59304(29) | 0.36025(24) | 0.70179(29) | 1 | 4.617(59) |
| C2 | 0.54000(25) | 0.43078(26) | 0.75990(36) | 1 | 4.617(59) |
| C3 | 0.5 | 0.5 | 0.70384(39) | 1 | 4.617(59) |
| C4 | 0.54135(26) | 0.42957(25) | 0.87919(26) | 1 | 4.617(59) |
| C5 | 0.5 | 0.5 | 0.93588(31) | 1 | 4.617(59) |

| | | | | | |
|---|---|---|---|---|---|
| H3 | 0.5 | 0.5 | 0.6267(31) | 1 | 5.541(71) |
| H4 | 0.57318 | 0.38162 | 0.90735 | 1 | 5.541(71) |
| H1 | 0.806 | 0.25 | 0.625 | 1 | 5.541(71) |
| C6 | 0.7595(20) | 0.5376(18) | 0.9408(18) | 0.3335(35) | 15.81(55) |
| O4 | 0.74263(99) | 0.46332(72) | 0.9319(11) | 0.3335(35) | 15.81(55) |
| O5 | 0.7765(11) | 0.6116(11) | 0.9480(15) | 0.3335(35) | 15.81(55) |
| C7 | 0.8943(64) | 0.7945(69) | -0.0750(61) | 0.1084(32) | 17.3(14) |
| O6 | 0.8774(37) | 0.7522(37) | -0.1539(60) | 0.1084(32) | 17.3(14) |
| O7 | 0.8951(29) | 0.8288(26) | 0.0123(59) | 0.1084(32) | 17.3(14) |

Atomic Coordinates for MFM-300(V)-300mbar$CO_2$

| Site | *x* | *y* | *z* | Occupancy | $B_{iso}$ (Å$^2$) |
|---|---|---|---|---|---|
| V | 0.690800(48) | 0.309200(48) | 0.5 | 1 | 3.550(25) |
| O1 | 0.74625(22) | 0.25 | 0.625 | 1 | 3.632(40) |
| O2 | 0.89849(15) | 0.28984(13) | 0.99324(22) | 1 | 3.632(40) |
| O3 | 0.62073(17) | 0.38260(16) | 0.39037(16) | 1 | 3.632(40) |
| C1 | 0.59003(29) | 0.36026(24) | 0.70240(30) | 1 | 4.538(58) |
| C2 | 0.54299(25) | 0.43307(26) | 0.76040(40) | 1 | 4.538(58) |
| C3 | 0.5 | 0.5 | 0.70380(40) | 1 | 4.538(58) |
| C4 | 0.54626(29) | 0.43256(27) | 0.87937(27) | 1 | 4.538(58) |
| C5 | 0.5 | 0.5 | 0.93619(32) | 1 | 4.538(58) |
| H3 | 0.5 | 0.5 | 0.6266(31) | 1 | 5.446(69) |
| H4 | 0.57318 | 0.38162 | 0.90735 | 1 | 5.446(69) |
| H1 | 0.806 | 0.25 | 0.625 | 1 | 5.446(69) |
| C6 | 0.7595(20) | 0.5376(20) | 0.9408(19) | 0.4194(50) | 20.45(61) |
| O4 | 0.74263(93) | 0.46332(66) | 0.9319(12) | 0.4194(50) | 20.45(61) |
| O5 | 0.7765(11) | 0.6116(13) | 0.9480(17) | 0.4194(50) | 20.45(61) |

| | | | | | |
|---|---|---|---|---|---|
| C7 | 0.8976(56) | 0.7904(55) | -0.0751(39) | 0.2793(49) | 32.7(12) |
| O6 | 0.8841(32) | 0.7497(26) | -0.1560(47) | 0.2793(49) | 32.7(12) |
| O7 | 0.9007(23) | 0.8301(18) | 0.0087(42) | 0.2793(49) | 32.7(12) |

Atomic Coordinates for MFM-300(V)-500mbar$CO_2$

| Site | *x* | *y* | *z* | Occupancy | $B_{iso}$ (Å$^2$) |
|---|---|---|---|---|---|
| V | 0.691230(50) | 0.308770(50) | 0.5 | 1 | 3.825(26) |
| O1 | 0.74470(22) | 0.25 | 0.625 | 1 | 3.740(41) |
| O2 | 0.89912(15) | 0.28965(14) | 0.99401(24) | 1 | 3.740(41) |
| O3 | 0.62005(17) | 0.38197(16) | 0.39104(17) | 1 | 3.740(41) |
| C1 | 0.59123(29) | 0.36045(25) | 0.70242(31) | 1 | 4.783(60) |
| C2 | 0.54327(26) | 0.43316(27) | 0.76017(42) | 1 | 4.783(60) |
| C3 | 0.5 | 0.5 | 0.70333(42) | 1 | 4.783(60) |
| C4 | 0.54678(30) | 0.43276(28) | 0.87918(28) | 1 | 4.783(60) |
| C5 | 0.5 | 0.5 | 0.93620(33) | 1 | 4.783(60) |

| | | | | | |
|---|---|---|---|---|---|
| H3 | 0.5 | 0.5 | 0.6260(32) | 1 | 5.740(72) |
| H4 | 0.57318 | 0.38162 | 0.90735 | 1 | 5.740(72) |
| H1 | 0.806 | 0.25 | 0.625 | 1 | 5.740(72) |
| C6 | 0.7595(17) | 0.5376(19) | 0.9408(17) | 0.4345(51) | 18.54(53) |
| O4 | 0.74263(83) | 0.46332(62) | 0.9319(11) | 0.4345(51) | 18.54(53) |
| O5 | 0.77654(97) | 0.6116(12) | 0.9480(15) | 0.4345(51) | 18.54(53) |
| C7 | 0.8976(58) | 0.7904(53) | -0.0751(35) | 0.3506(53) | 37.9(11) |
| O6 | 0.8841(32) | 0.7497(23) | -0.1560(45) | 0.3506(53) | 37.9(11) |
| O7 | 0.9007(23) | 0.8301(18) | 0.0087(40) | 0.3506(53) | 37.9(11) |

Atomic Coordinates for MFM-300(V)-750mbar$CO_2$

| Site | *x* | *y* | *z* | Occupancy | $B_{iso}$ (Å$^2$) |
|---|---|---|---|---|---|
| V | 0.691700(50) | 0.308300(50) | 0.5 | 1 | 3.594(26) |
| O1 | 0.74484(22) | 0.25 | 0.625 | 1 | 3.569(41) |
| O2 | 0.89838(15) | 0.28970(14) | 0.99408(24) | 1 | 3.569(41) |

| | | | | | |
|---|---|---|---|---|---|
| O3 | 0.61952(17) | 0.38196(16) | 0.39128(17) | 1 | 3.569(41) |
| C1 | 0.59118(29) | 0.35994(25) | 0.70153(32) | 1 | 4.702(61) |
| C2 | 0.54461(26) | 0.43393(27) | 0.76011(42) | 1 | 4.702(61) |
| C3 | 0.5 | 0.5 | 0.70346(43) | 1 | 4.702(61) |
| C4 | 0.54733(30) | 0.43311(28) | 0.87925(28) | 1 | 4.702(61) |
| C5 | 0.5 | 0.5 | 0.93621(33) | 1 | 4.702(61) |
| H3 | 0.5 | 0.5 | 0.6261(32) | 1 | 5.642(73) |
| H4 | 0.57318 | 0.38162 | 0.90735 | 1 | 5.642(73) |
| H1 | 0.806 | 0.25 | 0.625 | 1 | 5.642(73) |
| C6 | 0.2402(16) | 0.0376(17) | 0.3098(17) | 0.4575(48) | 18.91(51) |
| O4 | 0.25603(82) | 0.96293(56) | 0.3164(10) | 0.4575(48) | 18.91(51) |
| O5 | 0.22427(92) | 0.1122(12) | 0.3032(15) | 0.4575(48) | 18.91(51) |
| C7 | 0.2082(40) | 0.1067(41) | 0.0726(28) | 0.3885(45) | 32.83(85) |
| O6 | 0.2476(17) | 0.1259(22) | 0.1512(33) | 0.3885(45) | 32.83(85) |
| O7 | 0.1687(13) | 0.0875(17) | 0.9940(29) | 0.3885(45) | 32.83(85) |

Atomic Coordinates for MFM-300(V)-1000mbar$CO_2$

| Site | *x* | *y* | *z* | Occupancy | $B_{iso}$ (Å$^2$) |
|---|---|---|---|---|---|
| V | 0.69216(5) | 0.30784(5) | 0.5 | 1 | 2.89(3) |
| O1 | 0.7459(2) | 0.25 | 0.625 | 1 | 2.73(4) |
| O2 | 0.89899(16) | 0.29063(15) | 0.9925(3) | 1 | 2.73(4) |
| O3 | 0.61992(18) | 0.38223(18) | 0.39092(18) | 1 | 2.73(4) |
| C1 | 0.5922(3) | 0.3602(3) | 0.7003(3) | 1 | 3.86(6) |
| C2 | 0.5434(3) | 0.4332(3) | 0.7601(4) | 1 | 3.86(6) |
| C3 | 0.5 | 0.5 | 0.7033(5) | 1 | 3.86(6) |
| C4 | 0.5476(3) | 0.4332(3) | 0.8794(3) | 1 | 3.86(6) |
| C5 | 0.5 | 0.5 | 0.9362(4) | 1 | 3.86(6) |
| H3 | 0.5 | 0.5 | 0.626(3) | 1 | 4.63(8) |
| H4 | 0.57318 | 0.38162 | 0.90735 | 1 | 4.63(8) |
| H1 | 0.806 | 0.25 | 0.625 | 1 | 4.63(8) |
| C6 | 0.760(2) | 0.538(2) | 0.937(2) | 0.477(6) | 21.5(7) |
| O4 | 0.7415(10) | 0.4625(7) | 0.9302(13) | 0.477(6) | 21.5(7) |
| O5 | 0.7793(11) | 0.6127(13) | 0.954(2) | 0.477(6) | 21.5(7) |
| C7 | 0.207(4) | 0.100(4) | 0.075(3) | 0.453(5) | 32.9(9) |
| O6 | 0.2484(17) | 0.121(2) | 0.154(4) | 0.453(5) | 32.9(9) |
| O7 | 0.1672(13) | 0.0956(16) | 0.992(3) | 0.453(5) | 32.9(9) |

**6. Synthesis of PIM-1**

To a dry 100 mL round bottom flask equipped with a Dean−Stark trap and mechanical stirrer, 5,5′,6,6′-tetrahydroxy-3,3,3′,3′-tetramethyl-1,1′-spirobisindane (3.404 g, 0.01 mol), dicyanotetrafluorobenzene (2.001 g, 0.01 mol), anhydrous $K_2CO_3$ (4.146 g, 0.03 mol), DMAc (20 mL) and toluene (10 mL) were added under $N_2$. The reaction was heated to 165 °C and carried out for 35 min (15 min used to obtain equilibrium) under reflux. The resultant highly viscous solution was immediately poured into MeOH (500 mL) in order to quench the reaction. The product was then dissolved in $CHCl_3$ (150 mL) and re-precipitated from MeOH (1 L). The product was refluxed for 18 h in deionized water, filtered and washed with acetone and then dried at 120 °C for 2 days. This gave the desired PIM-1 product (3.84 g, 83% yield). GPC (in chloroform): $M_w$ = 230039 g mol$^{-1}$, and $M_w/M_n$ = 6.001. $^1$H NMR (500 MHz, $CDCl_3$, δ, ppm): 6.81 (2H, s), 6.43 (2H, s), 2.33−2.17 (4H, dd), 1.37−1.31 (broad, 12H). Anal. Calc. for C29H20N2O4 (wt %): C, 75.64; H, 4.37; N, 6.08. Found: C, 74.12; H, 4.24; N, 6.13.

## 7. PXRD of mixed matrix membrane MFM-300(VIII)/PIM-1

PXRD analysis of the membrane was conducted on a Philips X'Pert XRD using Cu Kα radiation to ensure retention of structure and crystallinity of the MOF once incorporated into the MMM.

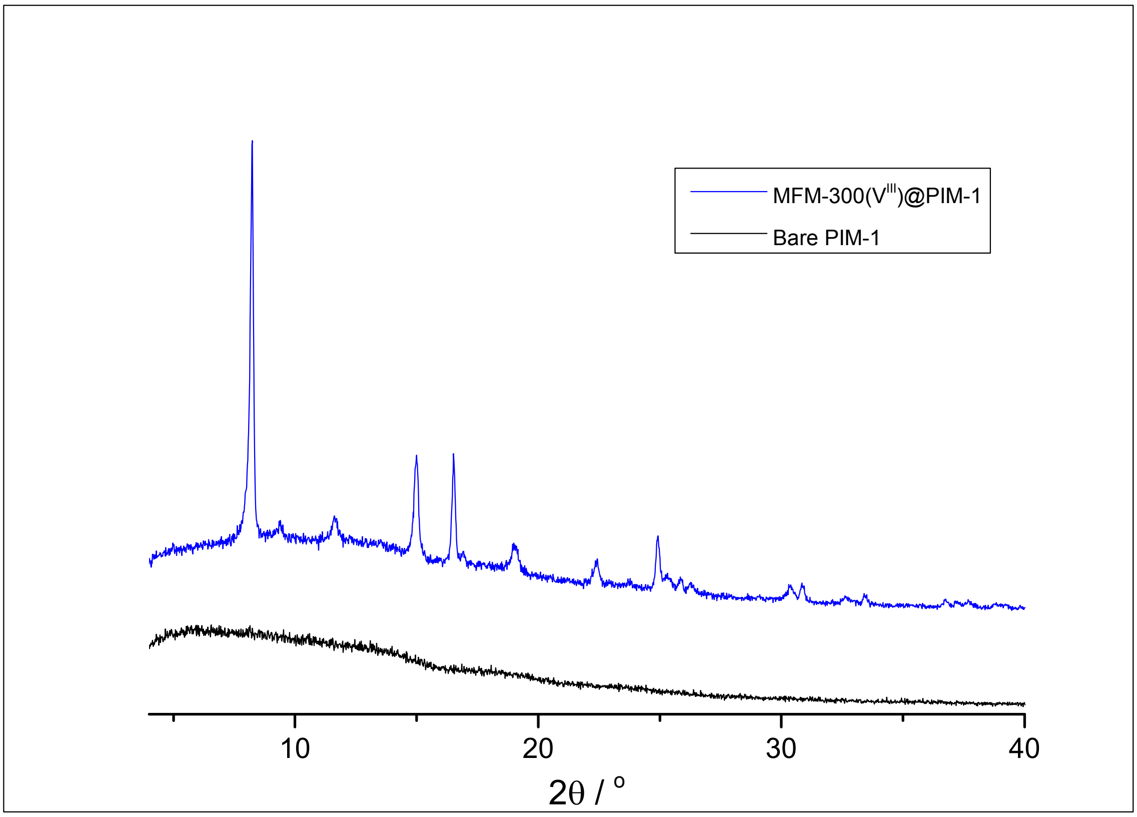


**Figure S15.** PXRD for PIM-1 and for MFM-300($V^{III}$)/PIM-1.

## 8. SEM of mixed matrix membrane MFM-300(VIII)/PIM-1

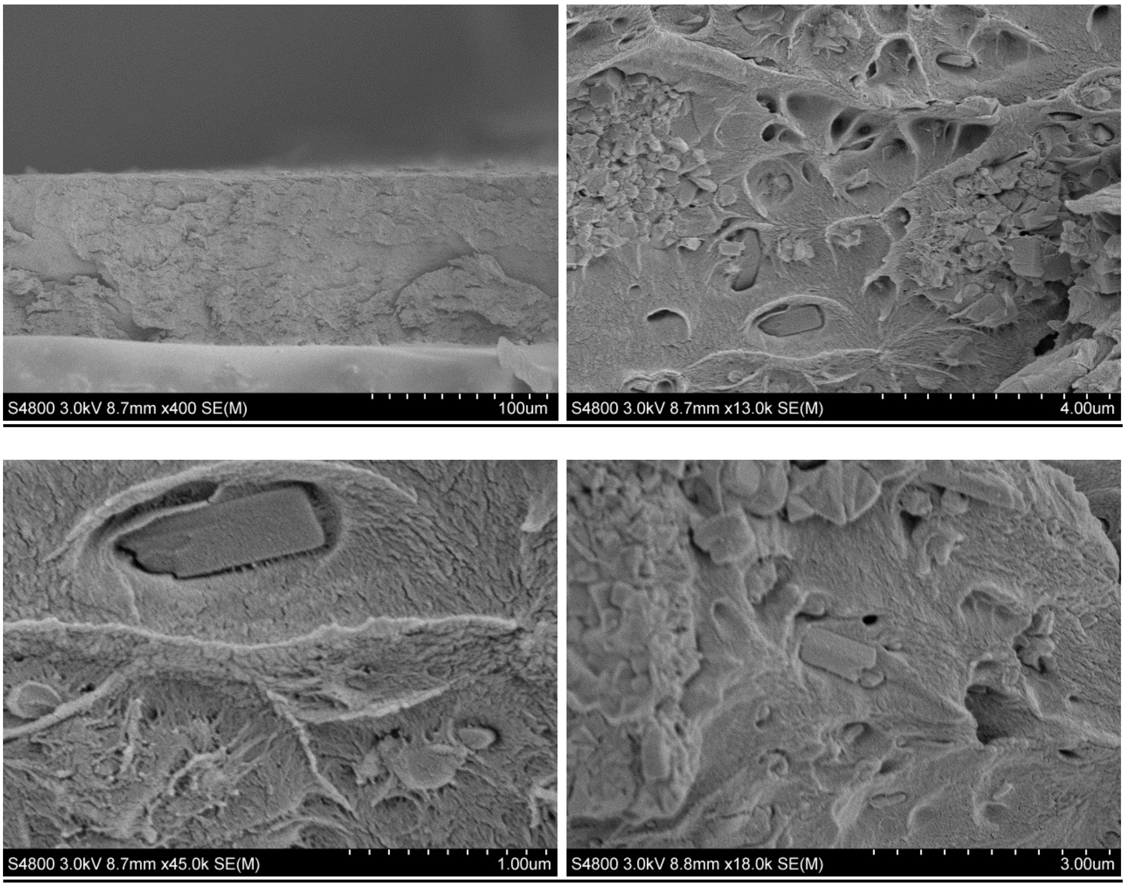


**Figure S16.** SEM images of the membrane cross-section showing good distribution of the MOF particles and good affinity between the particles and the polymer matrix.

## 9. Results of Permeation experiments

**Table S3.** Single gas permeation data of MFM-300($V^{III}$)/PIM-1 MMM.

| Gas | Temperature (°C) | Solubility coefficient ($cm^3$(STP) $cm^2$ $atm^{-1}$) | Diffusion coefficient (x$10^8$ $cm^2$ $s^{-1}$) | Permeability coefficient (Barrer) |
|---|---|---|---|---|
| He | 14 | 0.15 ± 0.01 | 6997 ± 451 | 1041 ± 2.45 |
| | 30 | 0.134 ± 0.008 | 8364 ± 562 | 1117 ± 1.35 |
| | 45 | 0.12 ± 0.0078 | 9819 ± 595 | 1191 ± 2.04 |
| | 61 | 0.11 ± 0.016 | 11816 ± 1720 | 1270 ± 3.90 |
| | 75 | 0.08 ± 0.008 | 16830 ± 1743 | 1379 ± 0.69 |
| $H_2$ | 14 | 0.56 ± 0.012 | 4190 ± 167 | 2342 ± 43 |
| | 30 | 0.458 ± 0.013 | 5381 ± 141 | 2465 ± 4.67 |
| | 45 | 0.40 ± 0.015 | 6414 ± 229 | 2542 ± 2.04 |
| | 61 | 0.33 ± 0.022 | 7894 ± 492 | 2611 ± 6.25 |
| | 75 | 0.26 ± 0.017 | 10697 ± 668 | 2758 ± 4.54 |
| $N_2$ | 14 | 2.0009 ± 0.057 | 79.38 ± 2.24 | 158.73 ± 1.94 |
| | 30 | 1.946 ± 0.008 | 111.39 ± 0.43 | 216.74 ± 0.52 |
| | 45 | 1.73 ± 0.012 | 158.14 ± 0.92 | 273.17 ± 1.12 |
| | 61 | 1.51 ± 0.006 | 216.98 ± 1.18 | 326.97 ± 1.99 |

| | | | | |
|---|---|---|---|---|
| | 75 | 1.33 ± 0.017 | 297.84 ± 2.46 | 403.33 ± 1.633 |
| **$CO_2$** | 14 | 58.58 ± 3.51 | 76.09 ± 3.13 | 4450 ± 83.84 |
| | 30 | 43.032 ± 1.57 | 107.37 ± 2.40 | 4617 ± 65.56 |
| | 45 | 31.22 ± 1.27 | 146.73 ± 3.80 | 4578 ± 67.69 |
| | 61 | 22.48 ± 0.54 | 196.84 ± 2.77 | 4423 ± 45.39 |
| | 75 | 16.92 ± 0.26 | 258.66 ± 1.82 | 4377 ± 37.05 |
| **$CH_4$** | 14 | 5.72 ± 0.30 | 34.36 ± 1.38 | 196.26 ± 2.65 |
| | 30 | 5.84 ± 0.07 | 51.53 ± 0.73 | 301.03 ± 0.41 |
| | 45 | 5.26 ± 0.065 | 79.15 ± 0.73 | 415.99 ± 1.30 |
| | 61 | 4.58 ± 0.068 | 117.87 ± 1.12 | 539.54 ± 2.99 |
| | 75 | 3.89 ± 0.041 | 175.98 ± 2.30 | 683.80 ± 2.09 |
